# Investigating belief understanding in children in a nonverbal ambiguous displacement and communication setting

**DOI:** 10.1016/j.jecp.2023.105830

**Published:** 2023-12-16

**Authors:** C.-N. Alexandrina Guran, Lucrezia Lonardo, Markus Tünte, Karla Arzberger, Christoph J. Völter, Stefanie Hoehl, Ludwig Huber, Claus Lamm

**Affiliations:** a Social, Cognitive and Affective Neuroscience Unit, Department of Cognition, Emotion, and Methods in Psychology, Faculty of Psychology, University of Vienna, 1010 Vienna, Austria; b Vienna Cognitive Science Hub, University of Vienna, 1010 Vienna, Austria; c Comparative Cognition, Messerli Research Institute, University of Veterinary Medicine Vienna, Medical University of Vienna and University of Vienna, 1210 Vienna, Austria; d Department of Developmental and Educational Psychology, Faculty of Psychology, University of Vienna, 1010 Vienna, Austria

**Keywords:** Theory of mind, False belief understanding, Nonverbal, Comparative research, Preschool children, Communication

## Abstract

Finding ways to investigate false belief understanding nonverbally is not just important for preverbal children but also is the only way to assess theory of mind (ToM)-like abilities in nonhuman animals. In this preregistered study, we adapted the design from a previous study on pet dogs to investigate false belief understanding in children and to compare it with belief understanding of those previously tested dogs. A total of 32 preschool children (aged 5–6 years) saw the displacement of a reward and obtained nonverbal cueing of the empty container from an adult communicator holding either a true or false belief. In the false belief condition, when the communicator did not know the location of the reward, children picked the baited container, but not the cued container, more often than the empty one. In the true belief condition, when the communicator witnessed the displacement yet still cued the wrong container, children performed randomly. The children’s behavior pattern was at odds with that of the dogs tested in a previous study, which picked the cued container more often when the human communicator held a false belief. In addition to species comparisons, because our task does not require verbal responses or relational sentence understanding, it can also be used in preverbal children. The children in our study behaved in line with the existing ToM literature, whereas most (but not all) dogs from the previously collected sample, although sensitive to differences between the belief conditions, deviated from the children. This difference suggests that using closely matched paradigms and experimental procedures can reveal decisive differences in belief processing between species. It also demonstrates the need for a more comprehensive exploration and direct comparison of the various aspects of false belief processing and ToM in different species to understand the evolution of social cognition.

## Introduction

The ability to attribute desires, beliefs, and intentions to the self and to other agents (be they human or nonhuman) is subsumed under the term ‘theory of mind” (ToM; Premack & Woodruff, 1978). ToM develops throughout childhood (see Rakoczy, 2022, for review) and includes the understanding of knowledge states (Moll & Kadipasaoglu, 2013) and differing beliefs of others (Wimmer & Perner, 1983). By 4 or 5 years of age, neurotypical children possess an explicit false belief understanding that they use to inform their interactions with others (Wellman et al., 2001). Indeed, understanding that others hold beliefs that can differ from one’s own is very important for a number of other social cognitive capacities (Rakoczy, 2022). False belief understanding also plays a role in deception understanding, which also develops around 4 to 6 years of age (Talwar & Lee, 2008).

It has been argued (but see, e.g., Bugnyar, 2017) that humans’ ToM skills, and how humans use them to guide their social behavior and build their expectations, are exceptional in the animal kingdom (Dunbar, 1998; Frith & Frith, 2005). Therefore, ToM has been a target of extensive research in other social animals, such as nonhuman primates (Heyes, 1998; Horschler et al., 2020; Kano et al., 2020; Krupenye et al., 2016; Lewis & Krupenye, 2022; Premack & Woodruff, 1978), dogs (Catala et al., 2017; Hare & Tomasello, 2005; Kaminski et al., 2009; Lonardo et al., 2021; Maginnity & Grace, 2014), and dolphins (Tschudin 2006, for a critical discussion, see Hill et al., 2018).

However, direct proof for ToM in other species, and even in children, can be elusive because of the concept’s strong linkage to language abilities (Wellman et al., 2001). In this study, we put children in a displacement task analogous to one we had previously used in pet dogs (Lonardo et al., 2021;) to investigate the children’s behavior and compare it with the behavior of the dogs.

Dogs are of particular interest for understanding the evolution of social cognition and behavior, including ToM. Dogs have outperformed great apes in their understanding of pointing gestures and their use of human gaze cues to inform their own behavior (Duranton et al., 2017; Gácsi et al., 2009; Miklósi & Soproni, 2006; Téglás et al., 2012; Wallis et al., 2015). This may be based on their phylogeny and a possibly selective breeding for understanding human social cues (Hare & Tomasello, 2005; Miklósi et al., 2004; but see Range & Marshall-Pescini, 2022). Indeed, in previous studies, pet dogs showed signs that they were considering the perspective of human communicators about the location of hidden food (Catala et al., 2017; Lonardo et al., 2021; Maginnity & Grace, 2014). The results of (Lonardo et al., 2021) even suggest that pet dogs are sensitive to the false or true beliefs of a communicator (see below).

However, there is no consensus about the presence of false belief understanding in nonhuman animals (but see Buttelmann et al., 2017, Kano et al., 2019, Krupenye & Call, 2019, Krupenye et al., 2016, and Lurz et al., 2022, for notable examples of nonhuman belief understanding research). In young children, a variety of tasks exist to investigate ToM and false belief understanding that can help to inform paradigms in nonhuman animals. In traditional displacement tasks (also known as change of location), such as the Sally-Anne test (Baron-Cohen et al., 1985; Wimmer & Perner, 1983), children are told about or shown a scenario in which an agent first puts an object in Location A, and the object is later moved to a Location B in the absence of the agent. The children are then asked to say in which location they think the agent will look for the object. Aside from relying heavily on language (Rakoczy, 2022), these ToM tasks often demand a high cognitive load from the children because they need to keep substantial amounts of information in working memory (Callejas et al., 2011; Mutter et al., 2006). Cognitive load is indeed inversely associated with ToM task performance (Schneider et al., 2012). Often, the tasks also demand strong inhibitory control in suppressing more automatic or common responses to favor less automatic ones (Southgate et al., 2007). Language, cognitive load, and inhibitory control are skills that either do not exist in or are particularly demanding for nonhuman animals.

To circumvent these issues, nonverbal measures have been developed and have helped outline the development of implicit or spontaneous forms of ToM (Rakoczy, 2022). In contrast to traditional ToM paradigms such tasks do not rely on language abilities and employ simpler settings in order to investigate more implicit and less demanding forms of mental state attribution (e.g., Buttelmann et al., 2009) given that they do not rely on children understanding relational sentences. Anticipatory looking paradigms that investigate whether participants take others’ mental states into account when observing a stimulus/event have shown that infants from 1 year of age (Southgate et al., 2007), but also non-human primates (Hayashi et al., 2020; Kano et al., 2019; Krupenye et al., 2016), show signs of implicit ToM. From 18 months of age, interaction-based tasks have shown that children take another agent into perspective by adapting their own behavior (Buttelmann et al., 2009). However, there is still a debate surrounding anticipatory looking paradigms for ToM research, and the replication of their findings (Kampis et al., 2021; Kulke et al., 2018; Schuwerk et al., 2021). In addition to differing developmental trajectories found for traditional explicit and implicit ToM tasks, recent neuropsychological evidence has also identified differing underlying brain networks in children (Grosse Wiesmann et al., 2020) and adults (Boccadoro et al., 2019).

Even when nonverbal and simpler tasks are employed, the translation to nonhuman animals is limited and rare (see examples above). Tasks are needed that can be applied in comparative research across different species with minimal adaptation. The current study addressed this challenge and pursued three objectives: (a) testing children’s spontaneous task performance in an ambiguous false belief setting without explicitly instructing the children with task goals, (b) comparing the performances of children and dogs in a task setting that was largely identical yet considered species-specific differences, and (c) validating a nonverbal approach to measure false belief understanding in children. Importantly, the canine data were collected previously and have been published elsewhere in detail (Lonardo et al., 2021).

Our task builds on a study by Call and Tomasello (1999), who found that children at 5 years of age were able to correctly choose the baited containers despite the misleading communicative cues in a false belief (FB) condition displacement task, whereas this was not the case for younger children and apes (see also Krachun et al., 2009, for a competitive task context). In Call and Tomasello’s (1999) study, participants were presented with a ‘finding game” where a reward was hidden by one adult human and its location was communicated by another. Of interest was the performance in a final false belief trial, where the communicating adult had not witnessed the hiding of the reward. In contrast to Call and Tomasello’s study), the dogs (Lonardo et al., 2021) and children in our study witnessed nearly all the hiding events (except for events in Phase 2 of familiarization) and were always privy to the locations of the stickers in the final test belief trials. This alteration was made because it is unclear whether dogs understand invisible displacements (Jaakkola, 2014). In addition, we also included a true belief (TB) condition that was missing in Call and Tomasello (1999).

In dogs, Lonardo et al., 2021 previously found response patterns that were not in line with common theoretical predictions based on false belief understanding in humans, namely disregard of cues when the communicator is not privy to reward locations, as is the case in FB trials. The majority of breeds (except for terriers) showed more cue following to the empty container in the FB condition compared with the TB condition, which was interpreted as the dogs possibly viewing the wrong cueing in the TB condition as deceitful, whereas the erroneous cue in the FB condition might have been considered an error ‘in good will.” Dogs may generally favor conforming to cooperative, reliable interaction partners (Dorey et al., 2010; Riedel et al., 2008), as in our study, especially in a situation with adult strangers. In general, dogs have been shown to engage in visual perspective taking (Catala et al., 2017; Kaminski et al., 2009; Maginnity & Grace, 2014) as well as to track human attentional states, and thus they have been interpreted to possess at least some basic building blocks of mental state attribution (see Huber and Lonardo, 2023 and Lonardo et al., 2021 for extensive discussion and review).

In this preregistered study, the children were not given any specific goal; they were only told to observe the adult and to pick one of the containers to look into in each trial. We used stickers in analogy to the food rewards used in dogs. This setup was chosen to make the paradigm as comparable as possible to our canine study and to investigate spontaneous task performance; whereas dogs are inherently motivated to find food rewards, children are inherently motivated to find stickers that are of high interest to them. We did not instruct the children to look for stickers; the dogs had not been specifically told to find rewards either and thus might have chosen their actions based on other additional considerations, such as a bias toward conformity with an adult (Marshall-Pescini et al., 2011; Prato-Previde et al., 2008), which may also motivate children in this task (Wilks et al., 2015).

We expected preschool children in the age range of 5 and 6 years to display some form of belief understanding that should inform their choices differently in the TB and FB trials. Children of this age should already possess a false belief understanding but were not yet entered into formal schooling (to ensure a better developmental homogeneity across the tested sample). Even presupposing ToM abilities, there are at least three possible response patterns in children with developed false belief or knowledge understanding: First, children may disregard the communicator’s suggestion in the FB condition more often than in the TB condition (see also Call & Tomasello, 1999). This would be in line with correct knowledge attribution and the interpretation of the communicator’s behavior in the FB condition as being caused by a lack of knowledge. In the TB condition, in contrast, the erroneous cueing by the communicator is less clear to interpret and might elicit a more random response pattern across the sample. Second, children may always choose the baited container and ignore the communicator’s cues irrespective of belief condition. This would be in line with their high motivation to obtain the reward (Logue & Chavarro, 1992) and the fact that they have the required knowledge to find it (because they witness all hiding events in the belief trials) as well as a disregard for others’ beliefs and knowledge. Third, children may selectively disregard the communicator in the FB condition but preferably select the cued container in the TB condition (Buttelmann et al., 2009), resulting in higher than chance rates of picking the cued container in this condition. This behavior would be in line with belief understanding accounts and the inference that the hitherto truthful communicator intends to show them something more relevant about the (empty) container in the TB condition.

Importantly, all but the second response pattern presuppose some ability of children to infer either knowledge states (first response pattern) or intentions (third response pattern) in the communicator. In addition to mirroring the study in dogs, by providing children with their own knowledge of the location of the reward, we can understand whether children act on inferred knowledge or inferred beliefs of the communicator and thus have a discriminatory marker of knowledge attribution or false belief understanding. Children who only take knowledge into account will disregard the communicator’s cue in both the TB and FB conditions. Children who presuppose some other, possibly helpful, intentions in the cueing of the empty container will follow this suggestion more in the TB condition than in the FB condition.

To ascertain whether preschool children in our sample possessed ToM abilities, we conducted a shortened version of the Wellman test battery (Kristen et al., 2006; Wellman & Liu, 2004) with all the children and a language understanding test (Grimm & Aktaş, 2001; not part of our preregistration) with a large subset of the sample. We also used these variables for further investigation, namely whether choices in the different belief conditions could be predicted by ToM abilities and language abilities, respectively. Given that our task was nonverbal, we did not expect language to have any effect. However, a link between higher ToM abilities and performance, especially in the FB condition, was expected. Likewise, we expected ToM abilities in our test to be predicted by age.

In addition to choice behavior, we also investigated the influence of belief trial condition (true vs. false belief) on latencies to retrieve the rewards. We expected children to be slower in the TB condition, where they had no readily available explanation for the experimenter cueing the ostensibly ‘wrong” empty container. Furthermore, we looked at whether children were faster when picking baited versus empty buckets. Note that the latency and language understanding analysis should be regarded as exploratory given that they were not part of our preregistration.

Finally, we compared the choice proportions of children and dogs statistically to be able to better understand the species differences in this goal-blind false belief task. The dog data came from the study by (Lonardo et al., 2021). If children showed similar behavioral response patterns as dogs, this would suggest that the task may tap into other less explored facets of belief understanding. However, as outlined above, we hypothesized that children would show clear evidence of false belief under-standing in this task by dismissing the cues in the FB condition more frequently than following them. If children possessing false belief understanding showed a different behavioral pattern than the dogs in the previous study, this may be interpreted as a weakening of the claim that dogs exhibit false belief understanding as measured by this task. However, it rather points to differing task demands and interpretations in the two species, or to additional factors other than the ToM capacities influencing an agent’s behavior in this task, such as a tendency to follow cooperative (human) adults.

## Method

### Participants

#### Inclusion criteria and recruitment

Initially, we recruited children aged 5;00 to 5;12 (years;months); however, due to COVID-19-related lockdowns in (Lonardo et al., 2021), data collection was heavily delayed. Therefore, we later opted to also test children aged 6;00 to 6;12 as long as they had not started primary school (which in (Lonardo et al., 2021) requires a minimum age of 6 years by September), a deviation from our preregistration. Children needed to be fluent in German, have normal or corrected-to-normal vision, and have no known neurological impairments or developmental delays. Families were recruited through a database of the developmental psychology lab at the (Lonardo et al., 2021), and all parents in this data-base with children in the targeted age range were contacted. Furthermore, the study was also advertised at a local kindergarten. Testing took place in testing rooms at the Faculty of Psychology of the Lonardo et al. (2021).

#### Sample

We based our sample size estimation on the power analysis conducted by (Lonardo et al., 2021) (Experiment 3), which revealed a power of 76% with 40 participants and an expected performance of .30 in the FB group and .70 in the TB group for a between-participants design. We finally recruited a total of 39 preschoolers (18 female) between April and December 2021. A total of 7 children needed to be excluded, 3 completely due to failing familiarization (1 girl; age range = 5.1–6.8 years) and 4 for the analysis of the second trial/GLM (generalized linear model) due to an experimentation mistake (sticker left in bucket in the first belief trial). The final sample for the generalized linear mixed model (GLMM) consisted of 32 children (16 girls) with a mean age of 5.56 years (range = 5.0–6.7 years).

#### Procedure

##### Experimental setup

**Fig. 1:**
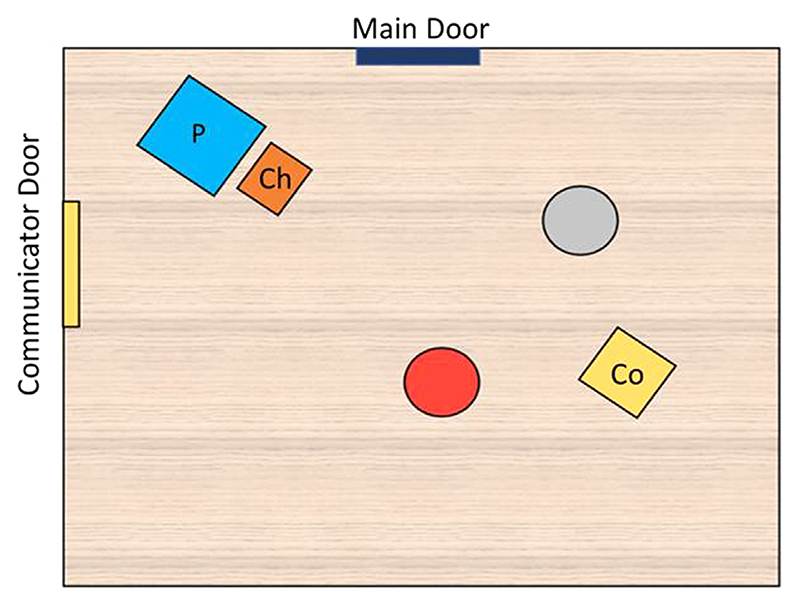
Experimental room setup. Colorful circles mark the location of the red and gray containers. P, parent; Ch, child; Co, communicator. (For interpretation of the reference to colors in this figure legend and of the baited and cued choices in Fig. 2, the reader is referred to the Web version of this article.)

The setup of the experimental room can be seen in Fig. 1. Our experimental setup was kept as similar to the dog study as feasible. Briefly, each child was seated on a pillow on the floor in front of the blindfolded parent (mother or father), who was sitting on a chair. Children were asked to face the experimental scene roughly 3 m in front of them, which consisted of two plastic buckets (red and gray) placed roughly 1.5 m apart (placement of all key objects was marked on the floor and therefore was identical for all children). Behind and equidistant to each bucket was a pillow to mark the crouching position of the communicator. The access to the room from the waiting area was through the main door. The hider and communicator always started or ended a trial by using the main door. The communicator door was exclusively used by the communicator during the final belief test trials. Setup and design of the experiment were kept as similar as possible to the dog study. Relevant changes are pointed out; some were necessary to ensure feasibility and ecological validity with young children.

##### Design

Our design was based on a paradigm introduced by Call and Tomasello (1999) and in analogy to Lonardo et al. (2021). Deviations from the dog study are pointed out where they arise. Participants were familiarized with different aspects of the belief trials in three phases of familiarization trials. In the first familiarization phase, children witnessed the hider entering the room and placing the reward (sticker) into one of the hiding containers and either leaving the room again (no displacement trial) or extracting the sticker from the container and displacing it to the other container before leaving the room (displacement trial). The placement and displacement of the sticker were done in an ostensive manner. If children were inattentive, the hider paused her actions until they resumed looking at the hiding. Upon the end of the trial (signaled by the cue word ‘okay”), children approached the containers and made their choice. This was identical to the dog study; however, it never happened that the dogs did not attend the food reward. Inattentiveness was also a rare occurrence in children. With this familiarization, children were introduced to the role of the hider, the process of hiding, and the possibility of finding rewards in the containers. Non-baited containers were empty. Children moved on to the next phase if they retrieved the reward in two consecutive trials (one with displacement and one without displacement where applicable) out of a maximum of four trials. The procedure was identical in the dog study, where a piece of food rather than a sticker had been used as a reward (all containers had been rubbed in food to ensure that smell did not influence the dogs’ choices).

The second familiarization phase introduced children to the role of the communicator. In this phase, trials started outside the experimentation room. The parent and child waited outside while the hider and communicator entered the experimentation room. The hider left the room again, and the parent and child were invited to re-enter the experimentation room. The child now watched the communicator cueing one of the buckets. The cueing procedure was standardized; the communicator left her crouching position and approached the baited container. She lifted the container and alternately gazed between the container and the child three times while saying ‘Look, this is good, this is very good” in French (*Regarde, ça c’est bien, ça c’est très bien!*). This cueing was the same as in the dog study, although in that study the verbal cue was given in English. Because many preschool children in Vienna, Austria received some formal or informal training in English, we used French as a language that none of the children spoke, understood, or were excessively familiar with. We asked all parents and participants whether they understood any French prior to the experiment. In the dog study, English was used to communicate with the participants; dogs’ understanding of semantic content of language is limited, of course, either to extraordinary individuals (Pilley, 2013) or to novel versus familiar word discrimination (Prichard et al., 2021). Still, language can offer useful information via tone of voice with or without semantic understanding (Andics et al., 2016). Given that the dog and human studies were conducted in Vienna, Austria, the dogs were generally not used to English. After this cueing, the communicator reverted back to her initial crouching position and gave the cue ‘okay” for the child to start to pick a container. Note that in this phase the children, as well as the dogs, did not witness the hiding of the reward and only had the communicator’s cueing as information. The criterion to pass in this phase was two consecutive correct bucket choices.

In the third familiarization phase, the full setup with both hider and communicator present was introduced to the children. As in Phase 1, children witnessed the hiding, but so did the communicator, who followed the actions of the hider in an ostensible manner. After the hider was done and had left the room, the communicator cued the baited bucket as in the second phase. Importantly, the communicator always cued the (correct) baited container during all familiarization trials in which she was present. The criterion for completion was the same as in Phase 1; the reward needed to be retrieved in two consecutive trials (one with displacement and one without displacement), out of a maximum of four trials, to proceed to the test trials.

After all familiarization phases were completed and passed, one of two belief trials followed (randomized order). In the FB trial, the hider and communicator entered the room together and took their places, and the hider hid the sticker in one of the containers. The communicator then left through the communicator door (see Fig. 1), and while she was gone the hider displaced the sticker to the other container and then left. The communicator came back into the room, through the communicator door, and proceeded to cue the empty container. In the TB trial, the hider and communicator entered together again, the hider hid the sticker in the presence of the communicator, and then the communicator exited through the communicator door again. While the communicator was gone (~15 s), the hider stood next to the container she had baited and waited. When the communicator came back and took her place, the hider proceeded to do the displacement of the sticker to the other container while both the child and communicator watched, and then the hider exited through the main door. Then, the communicator proceeded to cue the unbaited empty container. Importantly, the hider had covertly removed the sticker from the container during the final displacement in both belief trials so that no belief trials were ever rewarded.

In contrast to the dog study, FB and TB trials were administered within-participants with the human children participants, but each belief trial was preceded by a *complete* familiarization procedure on its own (all three familiarization phases, minimum two consecutive correct trials). We included a second familiarization with the second communicator for the children to learn anew that they should trust the communicator. This was a deviation from the dog study, where different trials were administered using a between-participants design. Thus, each dog got only one test trial in either a TB or FB condition. To ascertain that the wrong cueing in the first belief trial did not influence performance in the second belief trial, we used different communicators for each experimental block (performing the familiarization plus one belief trial). Thus, condition was a between-participants factor with the dogs, whereas it was a within-participants factor with the children. The total duration of the experimental trials never exceeded 30 min, including a break between blocks.

#### Instructions

Before the first familiarization phase, the experimenter gave written instructions to the parents and instructed the children verbally. Even though blindfolded, the parents were instructed not to interfere as well as to interact with their children as little as possible. In particular, they were provided standardized answers, such as ‘I am sure you are doing well” and ‘This sounds tricky indeed,” to answer potential questions from the children without biasing their choices. Finally, they were told that the experimenter would enter the room before some of the trials to give specific instructions, for example, before familiarization Phase 2 when participants were asked to stand up and follow the experimenter back into the waiting areas.

The verbal instructions for the children were framed as a game; the experimenter invited the children to play a game in which they could choose one of two containers placed in front of them. In each trial, the children could choose a container by standing up, approaching the container, and looking inside (each container was covered with a thick paper lid that matched the color of the container). The children were told that they could sometimes find something in a chosen container. If they found something, they were allowed to retrieve it and take it back to a box that their parents were holding. Although this framing as a game was a slight deviation from the dog study, it was necessary to ensure that children would indeed interact with the container setup in the room and is very commonly used in developmental research. We explained that the parents would sit behind them wearing an eye-covering mask; therefore, the parents could not answer questions due to them not seeing what occurred. When the children were unaccompanied in the experimentation room (*n* = 3), the box for holding the rewards was placed next to the children’s seating pillow.

The experimenter then introduced the three confederates (one hider and two communicators, one for each test block) as the child’s playmates, explaining that they would be playing with the child by entering the room and doing something with the containers in each trial. The children were asked to pay close attention to what the other playing partners were doing. Furthermore, the children were told that the confederates did not speak German (to prevent the children from trying to verbally interact with them). Finally, the children were told that their part in the game started after they heard the word ‘okay” stated by any of the play partners. This measure ensured that the children did not stand up before the hider and communicator were finished. In the dog study, the dog owners were blind-folded throughout the experiment while holding the dog and were instructed not to interact with their dogs except for releasing them upon the cue word ‘okay.”

#### Additional test materials

##### ToM test battery

We included two verbal ToM control tasks from a commonly used battery (Wellman & Liu, 2004; German translation: Kristen et al., 2006). Performance in these tasks was used as a covariate in the analyses. The first task was a location-related explicit FB task, where a small figurine was shown looking for his gloves, falsely assuming a wrong location for them. The second task was a content FB task, where the experimenter presented a Smarties box that surprisingly contained a small toy pig in the box. The children were then asked to judge what another agent (represented by another toy figure), who had never looked inside the box, would say was inside. The ToM control tasks and all other measures (see below) were administered after our FB displacement task. Of the final GLM sample, 22 children passed the content FB task (Smarties), 26 children passed the location FB task, and 18 children passed both tasks.

##### Language understanding for 3- to 5-year-olds

We assessed children’s language comprehension abilities with the SETK 3-5 (Language Development Test for Children Aged 3–5 Years; Grimm & Aktaş, 2001). This measure was not included in our preregistration because we had only considered it after the start of data collection. Hence, we have language ratings for only 24 participants (*M*_age_ = 5.54 years, *SD* = 0.58; 13 girls).

##### Questionnaire for parents

Effortful control enables children to suppress dominant responses and activate subdominant responses, to plan, and to detect errors (Rothbart et al., 2003). To investigate whether the ability for effortful control in children predicted performance in our false belief task, parents were asked to complete the very short form of the Child Behavior Questionnaire (CBQ; Putnam & Rothbart, 2006).

#### COVID-19 regulations and ethics

Our study was conducted during the COVID-19 pandemic. To ensure that everyone involved stayed as safe as possible, a few additional hygiene steps were taken: All adults, including parents, experimenters, and confederates, needed to present a valid negative COVID test upon arrival at the university (24-h validity for antigen, 48-h validity for polymerase chain reaction [PCR] tests). The communicators were vaccinated and also always had valid PCR tests because the procedure had them briefly remove their mask to be able to effectively communicate nonverbally with the children. The study was approved by the local ethics committee of the University of Vienna. The dog study had previously been approved and conducted at the Veterinary Medical University Vienna and complied with the ARRIVE (Animal Research: Reporting of In Vivo Experiments) guidelines (Kilkenny et al., 2010).

#### Analyses

We analyzed both children’s responses and their latencies until the responses. To analyze the main question of response patterns based on true and false belief, we looked at proportions of choice of baited versus empty containers in each condition. We planned to analyze both first touch and first choice (first container that was opened) responses separately; however, they were always identical. We analyzed response latencies from the ‘okay” verbal signal to the touch of a container.

Data were analyzed with (binomial) GLMM as well as with the parametric frequentist approach, and in the case of assumption violations nonparametric *t* tests, for more specific comparisons. To analyze the link between effortful control and language understanding, on the one hand, and choice performance in our nonverbal belief trials, on the other, we included the additional measures (CBQ and SETK scores) into our regression analysis in the GLMMs. Children who failed one of the ToM test battery subscales were not excluded from the main analysis, but ToM battery success was used as a covariate in the analyses.

In addition, we compared the dogs’ and children’s data using the binomial tests and by setting up a GLMM with participant ID as a random effect and species and condition as fixed effects/interaction.

## Results

### Children’s choice behavior

#### Binomial tests

**Fig. 2:**
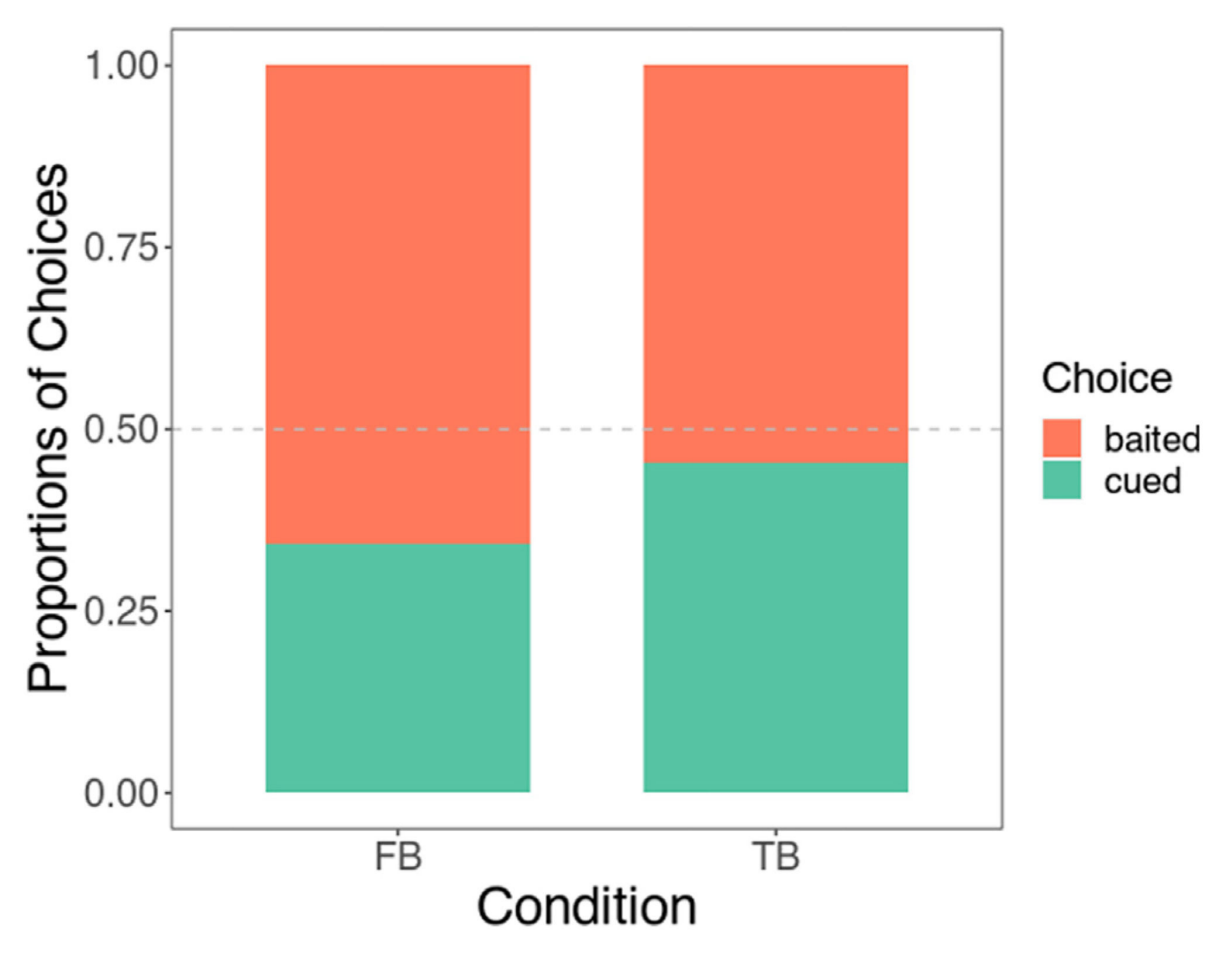
Proportions of container choices in each belief condition across the whole sample of children. FB, false belief; TB, true belief.

We compared choice proportions in each belief trial (TB and FB) with chance levels using binomial tests. In the FB condition, children picked the baited, not cued, container significantly more often than expected by chance (*p* < .05; 23 of 35 children, ~66%), whereas there was no difference from chance in the TB condition (*p* > .50; 18 of 33 children, ~55%) (see Fig. 2).

#### Generalized linear mixed model

**Fig. 3:**
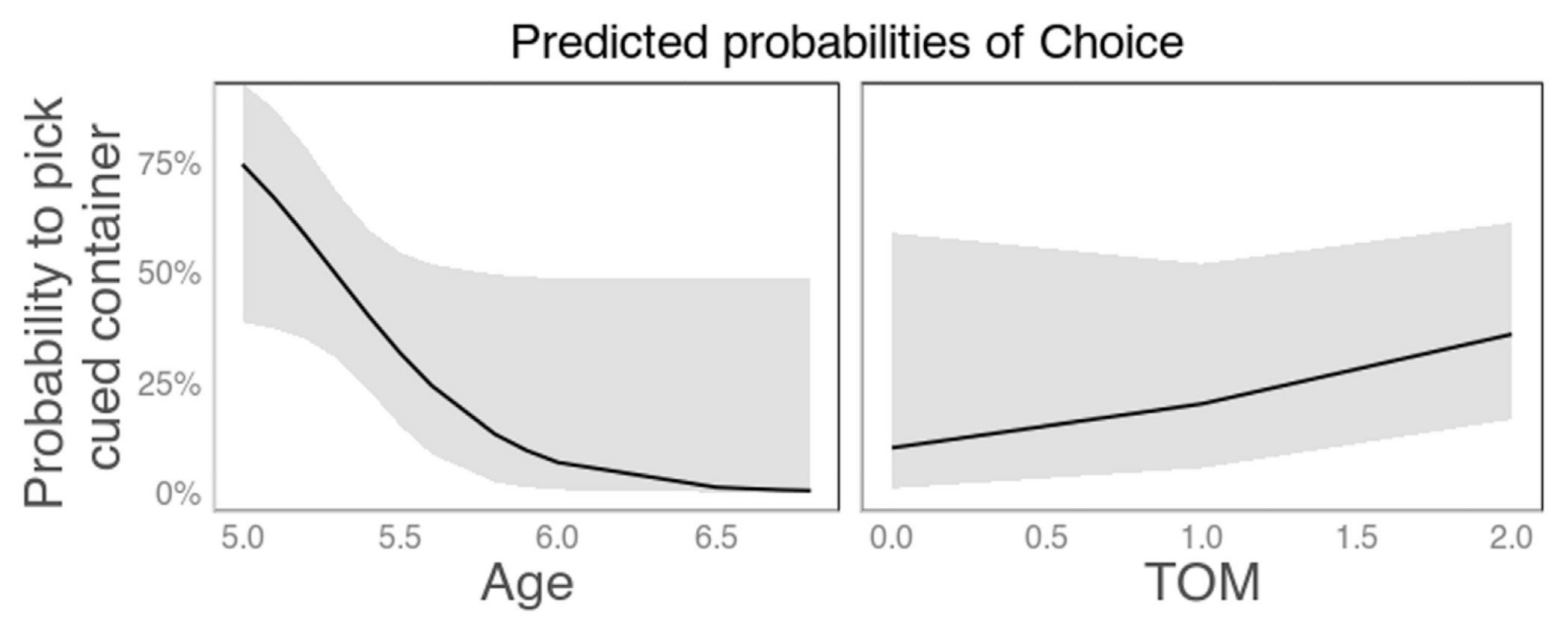
Probability of picking the cued container as a function of age and theory of mind (ToM) battery scores in children.

We analyzed choice patterns through a GLMM with 32 participants with participant as a random effect; condition, ToM scale points, age, and sex as fixed effects; and interactions with condition as well as a random slope of condition. We included fixed effects, interactions, and random slopes step by step in order of relevance to our hypotheses. The best model to outperform the null model included fixed effects of age (*β* = −3.71, *p* = .07) and ToM (*β* = 0.80, *p* = .22) as well as a random slope of condition and a random intercept of participant (*χ*^2^ = 11.779, Akaike information criterion [AIC] = 86.9, *p* = .02; however, note that both main effects failed to reach the significance level of *p* < .05). The probability to pick the cued but empty container (across both conditions) decreased with age and increased with higher ToM abilities as measured by the ToM test battery (see Fig. 3). In total, 2 children failed both ToM battery scales and 12 children failed one of the subscales. A model including condition as a main effect, but not as a random slope, performed worse (*χ*^2^ = 6.71, AIC = 86.94, *p* = .082).

#### Choice latencies

To ascertain whether differences between TB and FB trials influenced the time it took participants to choose a container, we conducted paired *t* tests between TB and FB choice latencies. Including correct and incorrect choices, participants seemed slower in the FB condition than in the TB condition (*M*_diff_ = 0.81 s), but this difference did not reach significance (*p* = .118, two-sided). When only accounting for correct choices (i.e., picking the baited container), a much reduced sample of only 10 participants could be analyzed in a paired *t* test and no significant difference could be found (including only participants who made correct choices in both conditions). When comparing latencies to pick cued versus uncued buckets with a GLM, we found no significant difference between correct and wrong responses (*p* > .90; *M*_error_ = 4.52 s, *M*_correct_ = 5.74 s).

#### Language understanding and CBQ scores

Because we did not have language understanding data for all participants, we ran a separate model with participant as a random effect; condition, ToM scale points, age, sex, and language understanding as fixed effects; and interactions with condition as well as a random slope of condition with a subset of 22 participants. These children (*M*_age_ = 5.6 years; 12 girls) on average reached the 65th percentile (range = 43–72). The best performing model (stepwise exclusion of fixed effects, stepwise inclusion of interactions and random slopes) in terms of AIC included a fixed effect of age (*β* = −4.11, *p* < .05), and a random slope of condition (model compared with null model: *χ*^2^ = 8.86, AIC = 61.66, *p* < .05). As in the analysis of the full dataset, older children chose more baited containers. In terms of the CBQ, there was no link between choice behavior and effortful control (all model *p*s > .60).

#### Comparing dogs and children

To investigate the performance of children and dogs, we compared the data of both samples by looking at results from separate binomial tests and by running a GLM that included both species (with participant ID as a random effect). Note that these analyses had not been preregistered because the preregistration had focused on analyses of the data from the children’s study exclusively. For context, in Lonardo et al.’s Experiment 1 (2021) Experiment 1, 29% of the 62 dogs in the TB group chose the empty container and 48% of the 58 dogs in the FB group did so, as compared with roughly 55% of children picking the baited container in TB and 66% doing so in FB (see ‘Binomial tests” section above).

The binomial tests showed interesting differences. In terms of relative risk (the equivalent of effect sizes for binomial tests), children were 1.31 times more likely to pick the baited container in the FB condition (significantly more likely than expected by chance; see ‘Binomial tests” section), whereas dogs were only 1.03 times more likely to do so. In the TB condition, children were 1.09 times more likely to pick the baited container, whereas dogs were 1.42 more likely to pick the baited container in this condition. The binomial test for FB in dogs did not reach significance (*p* > .40) but was significant for the TB condition (*p* < .001), suggesting that dogs pick the baited container more often than expected by chance in the TB condition.

Our model of interest, including species, condition and an interaction of the two, did not outperform the null model (AIC = 251.31, *p* = .14). However, the main effect of condition reached significance (*p* < .05). The main effect of species reached the trend level (*p* = .09), as did the interaction of species and condition (*p* = .06).

Using the findings from the binomial tests as well as estimated marginal means as a sort of ‘post hoc” test, we can unpack and interpret the GLMM results. Participants of both species were (some-what) more likely to pick the baited container than the empty container in the TB condition, but this was only significant in dogs. Finally, interpreting the trend level interaction, it seems that across all trials children are more likely than dogs to pick the baited container in the FB trials, whereas dogs are more likely than children to pick the baited container in the TB condition (disordinal interaction).

## Discussion

In this study, we investigated a nonverbal false belief displacement task in a sample of preschool children. The goal of this study was threefold: (a) to analyze children’s spontaneous task performance in an ambiguous false belief setting without explicitly giving task goals, (b) to compare the performances of children and dogs, and (c) to validate a nonverbal approach to measure false belief understanding in children.

Based on our analyses, children performed at random when choosing a container in the TB condition but disregarded the communicator’s misleading cue in the FB condition significantly more than expected by chance. In the FB trials, individuals with false belief understanding were expected to infer that the communicator may have suggested the wrong container because she did not know about the displacement, having been absent during the event. Thus, the results in the FB condition are consistent with the assumption that the children in our sample had developed a false belief understanding, or at the very least knowledge state attribution, which is in line with children’s (mostly successful) performance in the explicit ToM tasks we administered. This result is also in line with the existing literature showing explicit false belief understanding to emerge by around 4 years of age in neurotypical children (Grosse Wiesmann et al., 2017; Wellman et al., 2001).

However, true belief and false belief responses did not differ significantly between each other. TB conditions are often more difficult to interpret. Children may have interpreted the communicator’s cueing in the TB condition in different ways; the communicator might have been trying to show children something new and relevant about the container that had nothing to do with the presence of the reward or might have been suggesting the presence of another sticker that the children had not seen. In fact, it is possible that children in our study formed sophisticated hypotheses about the communicator’s views and intentions. At around 5 years of age, children begin to show second-order mental state understanding; that is, they reason about what other people know about others’ mental states as well as their own (e.g., ‘He knows that I know that .. .”; Sullivan et al., 1994; Talwar & Lee, 2008).

The fact that children picked the cued container numerically (i.e., in absolute numbers, not from the inferential statistics results) more often in the TB condition than in the FB condition lends tentative support to the idea that at least some children presupposed some other helpful intention in the communicator. If a majority of children had interpreted the communicator’s gesture as an attempt to deceive them, we would expect higher rates of cue disregard in the TB condition (similarly high as in the FB condition or higher; Ruffman et al., 1993).

In comparison with dogs, children’s performance in the FB condition can more readily be explained by what would be classically considered false belief understanding. More specifically, dogs in the FB group overall did not choose the baited container more often than dogs in the TB group, which would have been the classical pattern to be expected based on the literature on humans as well as on some nonhuman primates. However, dogs’ performance varied significantly between the two belief conditions, suggesting that dogs were sensitive to the different knowledge states of the communicator. Importantly, dogs (all breed groups except terriers, which performed as would be predicted for individuals with false belief understanding) picked the baited container more often than chance in the TB condition. Thus, the dogs’ reward-retrieving decisions may have been influenced by additional factors modulating the representation of the human communicator’s belief states. Such factors may include obedience toward humans or a conformity bias toward unknown interaction partners. These factors need to be investigated further.

Given that both species showed different responses between the TB and FB conditions (see results of binomial tests), we can conclude that the task is suited to ascertain the sensitivity to the different belief states of the communicators but might also carry some ambiguity and be influenced by additional considerations in dogs as well as in children.

Half of the child sample consistently picked the baited container in both belief conditions. This suggests a strong drive to find the reward regardless of ToM considerations. It is possible that these 16 children were solely motivated by the stickers and did not use the communicator’s suggestions at all when they had their own (conflicting) information. In the dogs, two thirds of the final sample chose the baited container in the trial they were presented with. This suggests a strong drive for the reward regardless of species. It is not possible to ascertain precisely whether this drive was stronger in one or the other species given the smaller human sample. However, the comparison between species seems to suggest that dogs were more likely to pick the baited non-cued container in general.

In terms of response latency, surprisingly, it seems that children may be slower in the FB condition (but note that the test just failed to reach significance). Although the difference was small, this might have had to do with slowed responses when children picked the baited container and thus needed to ignore the cueing, which is a very salient and thus difficult stimulus to ignore. This was corroborated by descriptively slower choice times for the correct choices of the baited container where the cue needed to be ignored, as compared with the incorrect choices of the cued container. Furthermore, the TB condition did not necessitate the children to represent the communicator’s conflicting knowledge state of the world and thus might have made it less complex as long as the children were not confused by the wrong cueing. Again, one should note that none of these effects reached significance.

In our GLMs, the factor of age seemed to play a role in explaining choice behavior. The older children were, the more likely they were to go for the baited containers and not the cued containers. Older children may be more or differently reward driven in their decisions (see Galván, 2013; but see also Vervoort et al., 2015). Alternatively, older children may have understood the possibility of deception by the communicator in the TB condition (Talwar & Lee, 2008). However, we did not find an interaction of age and condition (but an absence of evidence does not mean evidence of absence).

Indeed, the small sample size and each child doing one trial per condition limited the power of our study. We were constrained in sample size mainly by feasibility in terms of recruiting, which was complicated due to COVID-19 pandemic-related lockdowns in Vienna, Austria during 2021. Although our power analysis suggested a sample size of 40 children, this was based on the dog procedure where the belief trial factor was administered between-participants. Using a within-participants design increased our power. However, based on the results, the actual effect sizes may have been smaller than supposed in the power analysis. Other limitations of the study included the ToM test battery; to our surprise, we found that the children born mainly in 2015 and 2016 often seemed to lack knowledge of the candy ‘Smarties,” which was key to the successful passing of one of the two subscales used. It would be worth investigating whether other laboratories have had similar experiences, this was just a fluke of our sample, or it was a more specific issue of a German-speaking country. In addition, because we only looked at children who were likely to possess ToM and did not investigate those abilities extensively with a longer test battery, the variance in terms of ToM was much reduced, possibly preventing us from finding a significant effect of ToM in the GLM, whereas models including this factor have performed similarly well as our final model. A visual inspection of the trend in the data does indeed suggest that the likelihood of choosing the baited container increases with age (Fig. 3). Of note, a higher ToM score was linked to an increased likelihood of picking the cued container across both conditions. This may also be explained by interpreting the communicator’s behavior as indicative of intentions of the communicator to point out something that the children were agnostic to.

Neither language ability nor inhibitory control (as measured by the CBQ) played a role in our non-verbal false belief task. Importantly, the lack of an influence of language understanding on choice behavior underlines that using auditory communication without semantically understandable content for the children can be an easy cueing mechanism. This removed the need for children to be fluent in a language in order to investigate their ToM and related abilities in the current task. In addition to its already tested application in nonhuman animals, our task offers an additional avenue not just to investigate false belief understanding and ToM in younger preverbal children, where the presence of ToM-like abilities is still strongly debated, but also to investigate children that may be nonverbal or preverbal due to developmental delays.

We think that this adaptation of a false belief task holds promise for future research in the development of ToM-like abilities throughout neurotypical and atypical development as well as being a potential additional measure for nonverbal or preverbal individuals if systematically validated in those samples. Having nonverbal tasks that tap into ToM abilities, namely false belief understanding, is also very important for the investigation of ToM/false belief understanding in nonhuman animals. Our paradigm and approach, used in human children and dogs, could be a good starting point for investigating false beliefs in additional understudied species, such as herd animals who also live in social contexts.

### Considerations for comparative research

Although we designed this task in as close an analogy to the canine task (Lonardo et al., 2021) as possible, some notable differences needed to be made in order to make the task applicable to children. Verbal instructions were given to the children, including the information that the following procedure would be a game and that they were supposed to pay attention to the hider’s and communicator’s actions. The framing as a game is commonly used in developmental research (Hoehl et al., 2019). At that age, many children need to know what they are allowed to do in a strange setting in order for them to overcome their shyness and actually interact with the experimental setting. Thus, we also used this approach to keep ecological validity for children high. In addition, we told children to be attentive to the happenings. Although this may be seen as a deviation (in terms of instruction) from the dog study, note that the dogs’ intrinsic motivation to attend the scene and hiding procedures where a food reward was present was at ceiling. If a dog had been inattentive or distracted for a moment, the hiders would have paused their hiding procedure (as in the child sample). However, this was never necessary for any of the dogs in the dog study. Thus, we included this instruction in children to have them attend to the scene as much as possible and keep the attention level more similar to that of the dogs. Although these changes potentially might have contributed to the behavioral differences, they were made to keep a similar level of species-specific task requirement and difficulty between the studies. Furthermore, the studies were conducted with a between-participants design in dogs and with a within-participants design in human children. The rationale for a within-participants design in children was mainly to increase power given that recruiting children of a specific age range was limited by the available database and the constraints of a study performed during the COVID-19 pandemic. To reduce interference from the first belief trial onto the second belief trial, we switched communicators and re-ran the familiarization in the child study, thereby minimizing potential interference from one to the other belief trial.

## Conclusion

When left to make their own choices as to the goal of a task, half of the children will generally go for the reward regardless of the communicative cue, and this tendency seems to increase with the children’s age. However, children also take the beliefs of communicators into account when making their decisions of where to look for a reward, generally exhibiting understanding of what adults have and have not seen. In particular, children in our sample picked the baited container more often than chance in the FB condition but not in the TB condition. Thus, this nonverbal false belief task may be implemented with preverbal children, offering a further implementation of false belief tests in younger children and clinical samples. Whereas children show a choice behavior that is expected under the assumption that they possess a false belief understanding, dogs are able to discern between TB and FB conditions, but most breeds behave in opposition to human behavior patterns. Although one conclusion may be that dogs do not possess a ToM or belief understanding, the fact that they chose differently in the different conditions would render such an interpretation premature, in particular in light of the results found in terriers, which showed classical human false belief patterns. These findings, and the fact that dogs disregarded the cue in the TB condition significantly more than expected by chance, suggest that dogs may interpret the situation differently than children. We should ask ourselves whether canine ToM and belief understanding, or its additional considerations, might be *different* from human ToM. Anthropocentric views of animal behavior are limited (Bräuer et al., 2020; Wynne, 2004), and taking the animal’s ecology into consideration may lead to more conclusive results to understand animal minds (Buttelmann et al., 2017; Krachun et al., 2009). In conclusion, our study suggests that using closely matched paradigms and experimental procedures can reveal decisive differences in belief processing between species, in this case humans and dogs. More specifically, it allowed us to interpret not only the findings of our previous study in dogs as signs of belief processing, but also how similar, or rather how dissimilar, human children respond in a closely matched task. Going beyond the current research, this calls for a more comprehensive exploration and direct comparison of the evolution of the various aspects of false belief processing and ToM across the two species as well as in other species.

## Data Availability

Dogs’ and children’s data (without videos and anonymized) are openly available on GitHub. Analysis scripts are found in the GitHub repository as well (dog study: https://github.com/lonardol/fb_dogs; child analysis scripts: https://github.com/alexandrinaguran/CHOICES21; data available upon written request). The preregistration of the study can be found on AsPredicted (https://aspredicted.org/ng6ti.pdf). Data available online (github) for dogs, in aggregated format for children upon request (due to data security reasons)
